# Monkey Steering Responses Reveal Rapid Visual-Motor Feedback

**DOI:** 10.1371/journal.pone.0011975

**Published:** 2010-08-04

**Authors:** Seth W. Egger, Heidi R. Engelhardt, Kenneth H. Britten

**Affiliations:** 1 Center for Neuroscience, University of California Davis, Davis, California, United States of America; 2 Center for Neuroscience and Department of Neurobiology, Physiology, and Behavior, University of California Davis, Davis, California, United States of America; Rutgers University, United States of America

## Abstract

The neural mechanisms underlying primate locomotion are largely unknown. While behavioral and theoretical work has provided a number of ideas of how navigation is controlled, progress will require direct physiolgical tests of the underlying mechanisms. In turn, this will require development of appropriate animal models. We trained three monkeys to track a moving visual target in a simple virtual environment, using a joystick to control their direction. The monkeys learned to quickly and accurately turn to the target, and their steering behavior was quite stereotyped and reliable. Monkeys typically responded to abrupt steps of target direction with a biphasic steering movement, exhibiting modest but transient overshoot. Response latencies averaged approximately 300 ms, and monkeys were typically back on target after about 1 s. We also exploited the variability of responses about the mean to explore the time-course of correlation between target direction and steering response. This analysis revealed a broad peak of correlation spanning approximately 400 ms in the recent past, during which steering errors provoke a compensatory response. This suggests a continuous, visual-motor loop controls steering behavior, even during the epoch surrounding transient inputs. Many results from the human literature also suggest that steering is controlled by such a closed loop. The similarity of our results to those in humans suggests the monkey is a very good animal model for human visually guided steering.

## Introduction

Guidance of locomotion is a fundamental behavioral ability for every motile organism. For primates and other terrestrial vertebrates, the most important sense guiding locomotion is visual. Locomotion, however, is typically a complex behavior involving numerous body parts and muscles, making it difficult to analyze at a physiological level. We are principally interested in the sensory aspects of the guidance of locomotion, and in particular their physiological substrates. In order to understand the physiological processes underlying sensory integration for locomotion, it is desirable to develop a simplified animal model that captures the essential aspects of locomotion. Steering using a single-dimension control, such as a steering wheel, has been extensively studied in humans. It is clear that numerous visual and non-visual cues can contribute. Estimates of the egocentric location of the target, current trajectory from optic flow, and extra-retinal cues are all proposed to mediate accurate steering [1], [2], [3], . However, despite extensive work, fundamental questions remain unanswered about steering. One of the most significant unsettled controversies concerns whether or not stereotyped steering adjustments, such as lane changes, are performed open-loop, using a “snapshot” of the steering error, or in a more closed-loop manner, constantly updating the error signal. Observers can perform steering adjustments quite accurately even in the face of substantial occlusion of visual input [6], yet other studies suggest continuous monitoring of steering error [2], [7]. We have developed a new method to address this question, exploiting the extensive data sets that can be collected from nonhuman primates in the laboratory setting.

We trained macaque monkeys to pursue a visual target as it moved in a virtual environment using a single axis joystick. The monkey's task was to control its trajectory across a textured ground plane so that it was on path to the target. While the monkey was maintaining heading, we would randomly displace the target to a new direction, forcing the monkey to make rapid steering movements to continue obtaining reward. The monkeys learned this task rapidly and well, and their behavior was quite similar across individuals. In this paper, we quantitatively characterize these rapid steering movements and relate them to stimulus parameters. In our task, two visual cues were always present and concordant: the target position and the optic flow. In this work, we focus on the dynamics of the behavior, and are agnostic about the weights being given to the available cues. This question will be addressed in future work.

As in humans, the size and duration of the steering response was highly dependent on the size of the target step, but the responses also exhibited considerable variability. Examination of the variability in the response over time suggests that a substantial fraction of the steering response is predicted by steering errors in the recent past. This observation is only consistent with a closed-loop model of steering control.

## Materials and Methods

### Hardware and software

Stimuli were generated on a dedicated computer by custom software (written by A.L. Jones and D.J. Sperka) that used OpenGL libraries running under a real-time Linux kernel. The display computer communicated with the experimental control computer over a dedicated Ethernet connection. The experimental control computer ran Rex, the NIH public domain package developed at the LSR, NEI. The joystick was analog and sent a DC voltage to the control computer, linearly related to the deflection of the joystick. This voltage was sampled at 1 KHz by a 12-bit ADC. The joystick position linearly controlled the angular velocity of the trajectory and was updated at a frame rate of 85 Hz.

Monkeys would sit in a darkened room, their views centered on a computer monitor running at 85 Hz with a resolution of 1024×768 pixels and maximum luminance of 60 cd/M^2^. The darkened room minimized the use of the monitor outline as a position reference frame. The monitor was viewed at a distance of 28 cm for monkeys F and J and 35 cm for monkey M, giving a field of view of 60° horizontally by 45° vertically for all monkeys.

**Figure 1:**
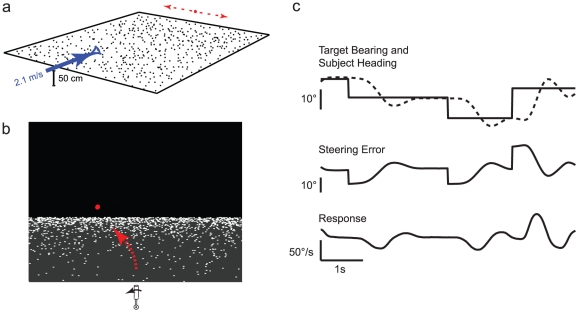
Task design and geometry. a) Schematic of the steering task. The monkey viewed the scene from the position of the blue arrow, which was 50 cm above the simulated ground plane. In our task, the monkey translated at a constant speed of 2.1 m/s across the ground plane, but could control its yaw. The monkey's goal was to turn until its current trajectory was toward the target (red dot). b) Scene as viewed from the primate chair. Upon seeing the target stepped from the center of the screen, the monkey was trained to correct its trajectory so that it would follow a path that led to the target (red dot). The red arrow illustrates one possible path to the target. c) Example traces from a single steering epoch. Top: target bearing (solid) and subject heading (dashed) over time; bearing and heading are relative to an arbitrary reference frame. Middle: steering error over time. Steering error is defined as the target bearing relative to the subject's heading. Bottom: Steering response over time.

Monkeys viewed a screen containing a 0.25° diameter red target dot just above the virtual horizon (see figure 1b). The ground plane dots were 0.1° in size, not scaled with virtual distance, and approximately 2500 were in view at any given time. Their luminance was 60 cd/m^2^, on a background of 7 cd/m^2^ to reduce persistence aftereffects. If one considers a virtual camera height of 50 cm (about the height of a monkey), the speed of the virtual translation was 2.13 m/s. There are many other visually identical geometries, of course, that differ only by a scale factor. Scaled to a typical human viewing height of 170 cm, the forward speed would be approximately 7.7 m/sec: a fast running pace. This relatively high speed was intended to increase the srength of the optic flow cue, and to reduce the effects of small eye movements around fixation. Movements of the joystick would cause a turn to occur, and there was a fixed scaling between stick deflection and angular velocity. For two of our monkeys (F and M), the scale factor was set so that maximum stick deflection would produce a turn of 255°/s; for monkey J it was set to produce a turn of 85°/s. The field of view was yoked to the current heading, like the field of view through the windshield of a moving car. Therefore, steering errors manifest themselves in two ways: the steering target is off-center on the screen, and the optic flow no longer aligns with the target. Just as in natural steering behavior, two concordant visual cues were always present in this task.

### Task and training

Two adult females and one adult male rhesus macaque (*Macaca mulatta*) were used in this study. All were trained to steer using positive reinforcement operant conditioning techniques. While sitting in an enclosed primate chair (Crist Instruments), monkeys were trained to extend a hand through a slot and maneuver a single-axis joystick that was attached to the outside of the chair. They were then trained to steer by rewarding them for maintaining their trajectory within a specified window around the current target position. Initially, this window was generous, but was reduced gradually to an asymptotic value of 3°.

Prior to data collection, monkeys were surgically implanted with a head restraint post (Crist Instruments) and a scleral search coil [8], under deep anesthesia using sterile technique. All animal procedures were in accordance with the ILAR Guide for the Care and Use of Laboratory Animals and were approved by the UC Davis IACUC.

The paradigm began with the target dot centrally located on the horizon and stationary for 500 msec before stepping to the left or right of the current target position by a specified amount. At this point, the groundplane would start to move and the monkey could steer with the goal of bringing the target back to the center of the screen, in line with the current trajectory. Thereafter, steps of random directions and specified amplitudes were presented, with intervals chosen from a clipped exponential distribution (1000 ms minimum, average 2000 ms). The amplitudes ranged from 5–25°, but with probability of occurrence weighted unevenly, with maximum probabilites for the lower-amplitude steps, according to a Gaussian probability distribution. However, the range of step sizes varied from day to day. See Table 1 for the total number of steps contributing to the data analyzed for this paper. Each trial would last for about 15–30 seconds of continuous steering, and trials werre separated by a 2-s interval. Such trials were presented in blocks of 10–60 trials, in which balanced numbers of all steps were presented.

**Table 1:** Numbers of target steps used for analysis.

					Jump size					
subject	−25	−20	−15	−10	−5	5	10	15	20	25
**F**	381	1781	2963	7628	1928	2065	8783	3185	1970	440
**J**	174	1193	1338	5547	2468	2507	5672	1327	1278	180
**M**	337	1958	2691	8344	2139	2260	8831	3146	2064	431

### Fixation

Most of the data we analyze, the animals were free to move their eyes at will. We also trained monkey F to steer while fixating a stationary green target 0.25° in diameter at 10 different fixation locations (X, Y, respectively: −20°, 20°; −20°, 2°; −5°, 20°; −5°, 2°; 0°, 20°; 0°, 2°; 5°, 20°; 5°, 2°; 20°, 20°; 20°, 2°). To complete a trial, the monkey needed to keep fixation within 1–2° of the target the entire duration of the steering bout. If the monkey failed to maintain fixation, we removed the target steps from that trial from analysis. To limit the data needed, we used only two target step amplitudes, 10° and 20°. All other aspects of this task were the same as the free-gaze condition.

### Data collection and analysis

Eye position and the raw joystick response were sampled at 1 MHz. However, since our video feedback was at 85 Hz, we stored and analyzed at this frequency, using the samples that were used for video updating. Data were analyzed in windows around each step of the target, excluding epochs where the monkey was not moving the joystick for 2 seconds. We also excluded data from trials where the monkey's root mean squared steering error (angular separation between current trajectory and target) was greater than its average error by 1 standard deviation. The number of steps contributing to the analysis for each step size is listed in Table 1.

**Figure 2:**
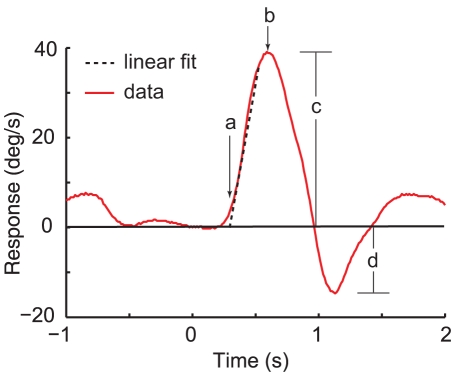
Parameters extracted from a step response. a) latency, b) time to peak, c) peak amplitude, d) amplitude of the overshoot correction.

To further examine the steering response data, we parametrized each steering response by its latency to the initial response, slope of the rising phase, peak of the response, the time to the peak response, and the amplitude of the overshoot correction (Figure 2).

### Latency and slope of the initial response

To find the latency to the initial response we fit the steering responses in a window after each step to a simple linear expression:
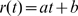
(1)


The time window used for the regression was 294–529 ms for monkeys F and M, and 294–553 ms for monkey J. Using the slope and intercept from each trial, we calculated the latency as:
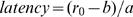
(2)Where *r*_0_ is the mean response of each monkey in a window 12–118 ms after all target steps and was interpreted as the baseline for the response.

The slope for each trial was taken as *a* from equation (1). Because the slope is the change in velocity over some small window in time, *a* approximates the angular acceleration of the monkey towards its target.

### Peak response and time to peak

We took the peak of the response to be the largest steering response in the direction of the target in the from 294–1177 ms after the target step. The time of this value, relative to the time of the target step, was taken as the time to the peak response.

### Overshoot correction

Similar to the peak response, we took the largest amplitude steering response in the direction *opposite* of the initial target step in a window from 706–1647 ms to be the amplitude of the overshoot correction on individual trials.

Some trials from the above analysis were excluded from further analysis because they fell outside of biologically plausible ranges. No more than 10% of the data were discarded for any one parameter.

### Correlation analysis

To examine feedback to the steering system, we performed an analysis of the correlation between the steering error and steering response across all trials for a given target step size. Therefore, we calculated the correlation coefficient between the two signals at times *t* and *t'* as:
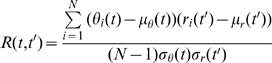
(3)Where *θ_i_*(*t*) is the steering error at time *t* for the *i^th^* step response, *μ_θ_*(*t*) and *σ_θ_*(*t*) are the mean and standard deviation of the steering error across all *N* responses to a given step at time *t*; similar conventions were applied to the steering responses, *r_i_*(*t*). The correlation coefficient was calculated for all combinations of times *t* and *t'*, and were displayed as a matrix image.

## Results

### Mean step response

**Figure 3:**
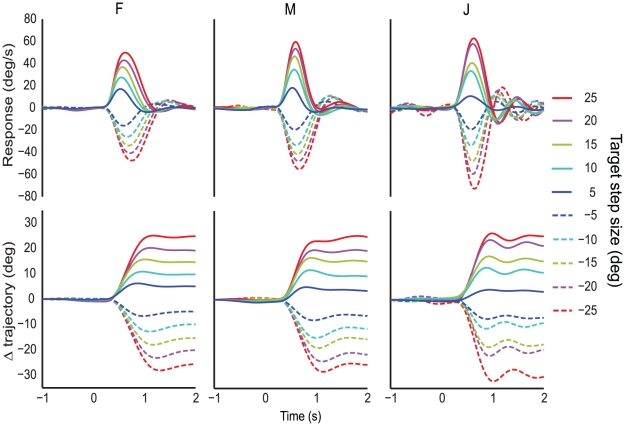
Mean response and change in trajectory for a target step. Top) Mean steering response in degrees per second to a target step from −25° to 25° for monkeys F, M and J. Bottom) Mean change in trajectory during a step response.

We trained three monkeys to manipulate the joystick to control their trajectory across the virtual ground-plane. The general shape of the steering response to a displacement of the target from the current trajectory was similar for all three monkeys (Figure 3). Approximately 250–350 ms after the target steps to its new location, the monkeys initiate a turn toward the target by moving the joystick in the direction of the target. As the response evolves, the monkey's turn accelerates towards the target, reaches a peak velocity and then begins to decelerate as the monkey's trajectory comes into line with the new target direction. The monkeys tend to oversteer, however, giving rise to a damped oscillation in the steering response as the trajectory converges with the target. This similarity in the general shape of the response suggests the steering strategy and the underlying “steering system” is similar across experimental animals. However, minor individual differences in the shape of the responses suggest parametric differences between subjects.

Examination of the mean steering responses to different amplitude target displacements reveals that the exact shape and time course of the steering response varies depending on the amplitude of the displacement. The latency to the initiation of the steering response appears similar across all step sizes However, the responses quickly diverge as time goes on, because the amplitude of the initial acceleration scales with the size of the target step. Similarly, the amplitude and time to the peak turn velocity also depend on the amplitude of the target step. As the new trajectory is achieved, the responses converge, becoming less dependent on the amplitude of the target step. These observations suggest that, in early phases of the response, the monkey's turn depends on the initial magnitude of the steering error; over time, however, the size of the target step becomes less important to the steering response. However, averaging the response over time could mask a more complicated process by smoothing across the individual responses. Therefore we analyzed the individual response parameters of all three monkeys to examine steering responses on a trial by trial basis.

### Latency

**Figure 4:**
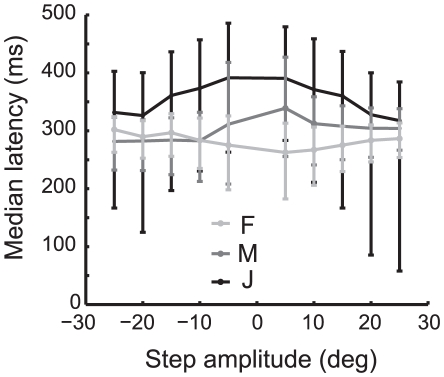
Latency to the initial response. Median latency by monkey and step amplitude. Error bars span the second and third quartiles of the data.

As noted above, the mean response latency to a large target displacement appears very similar across displacement amplitude. To explore this further, we fit a line to the rising phase of the response and took the time at which the line crosses the perceived zero point in the response as the latency to the response for each trial (Figure 2; see methods for details). The median latency to response is roughly similar across step amplitudes (Figure 4), although there were modest-amplitude dependencies on the step size. A two-way analysis of variance revealed a significant effect of both monkey and target step amplitude (amplitude: F_df = 9_ = 149.87 p-value<0.001; monkey: F_df = 2_ = 946.62, p-value<0.001). With the exception of monkey F, the latencies increase as the amplitude of the target step is reduced (figure 4). We suspect that this is at least partly a consequence of noise in the baseline. The RMS steering error between steps was 3.91°, 6.50°, and 11.37° for monkeys F, M and J, respectively. The animal with the lowest noise in her steering showed the least amplitude dependence, supporting this view.

### Initial slope

**Figure 5:**
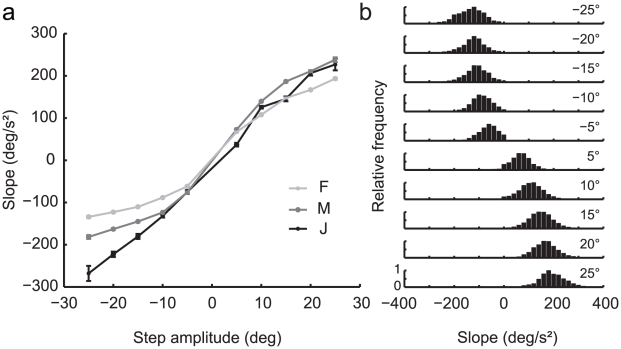
Initial acceleration as a function of step amplitude. a) Mean slope of the initial response by monkey and step amplitude. The slope of the response corresponds to the initial acceleration of the monkey's turn (see Methods). Error bars represent the standard error of the mean. b) Distribution of slopes for monkey F by step amplitude. Similar results were found for monkeys J and M.

Figure 3 shows a systematic increase in steering response amplitude as the step size increased. Greater steering response was clearly acheived primarily by larger turn amplitudes, rather than by increasing the duration of the turning movement. To characterize this increased acceleration in larger turns, we fit lines to the initial part of the turn (Figure 2; see methods). Indeed, the mean slopes scaled monotonically with target step amplitude (Figure 5a). Two of the monkeys showed a modest roll-off of response, particularly for left turns. These are not imposed by either the task or the hardware, and might be usefully correlated with physiological measurements in future experiments. There was considerable variation in the initial acceleration from trial to trial. Still, the distribution of the slopes for each target step was approximately Gaussian about each mean (figure 5b), suggesting the initial acceleration is a product of the size of the target step plus approximately constant noise.

### Peak of the response

**Figure 6:**
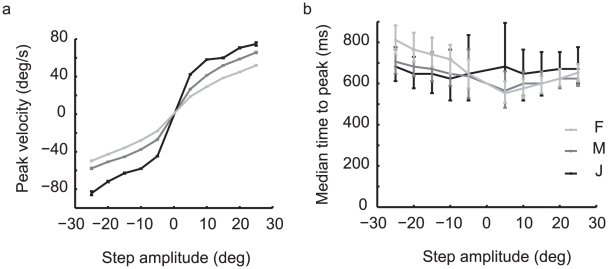
Peak response to a target step. a) Mean peak velocity by monkey and step size. Error bars represent the standard error of the mean. b) Median time to the peak response by monkey and step size. Error bars span the first and third quartiles of the data.

Consistent with greater acceleration, the maximum turn amplitude also increased strikingly with step size. Figure 6a shows the nature of this relationship, which was nearly linear, only exhibiting modest saturation. There were also modest changes of the timing of the peak with step size. As can be seen in figure 6b, the median time to the peak response increased with the absolute amplitude of the response in monkeys F and M. The trend is not as strong in monkey J, but the peak in the response to small steps may be occluded by the noise in monkey J's steering. A one-way analysis of variance reveals the effect of the amplitude of the target step on the time to the peak response to be highly significant (monkey F: F_df = 9_ = 1915.51, p-value<0.001; monkey M: F_df = 9_ = 186.81, p-value<0.001; monkey J: F_df = 9_ = 13.69, p-value<0.001). These differences in the time to the peak by step size do not correlate with differences in the response latencies found above. However, the increased time to the peak does relate to the acceleration and peak velocity of a response; the roll-off in acceleration (Figure 5) results in an increased time to the peak velocity.

### Overshoot correction

**Figure 7:**
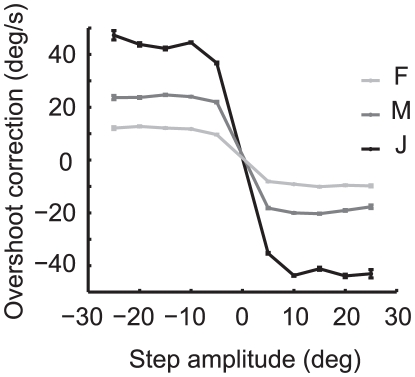
Mean velocity of the overshoot correction by monkey and step size. Error bars represent the standard error of the mean.

Following the peak of the monkey's response, the monkey decelerates its turn and usually reverses its steering, having overshot the target bearing. In the mean response traces, responses to target steps in the same direction tend to converge (Figure 3). To examine this on a trial-by-trial basis, we took the maximum response in the direction *opposite* of the target step in each response trace to be the overshoot correction amplitude. These values are shown in Figure 7. While the overshoot following target steps in the same direction significantly differed (ANOVA p-values were <0.001 for all monkeys in both directions), the overshoot amplitude differed by less than 25% for responses to steps in the same direction. However, there were large and consistent differences between monkeys. Clearly, the damping of steering movements is the most variable part of the behavior we observe in our task, and probably reflect differences in steering “tactics” across animals.

### Correlation analysis

One view of human steering is that it results from a continuous visuo-motor loop [2], [7]. In such models, the current estimate of steering error is transformed after a processing delay into a motor response leading to a change in the steering error and a new error estimate, closing the loop. If such models are correct, we expect the steering responses to correlate with recent estimates of the steering error. To test this hypothesis, we examined the correlation over time between the response and the steering error (see methods). The correlation matrix displays the correlation coefficient, time point by time point, between the steering error and the response. If steering behavior is the product of a visual-motor loop with a constant lag, a diagonal lobe of positive correlation should appear parallel to and below the unity line.

**Figure 8:**
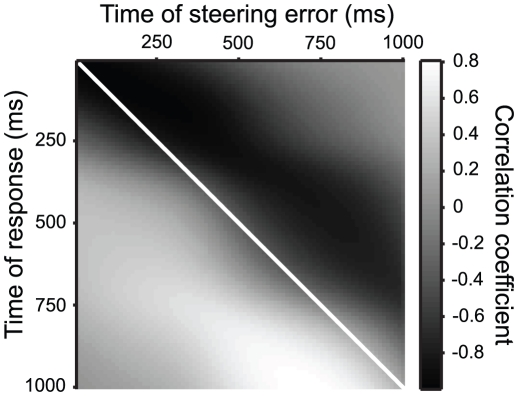
Correlation matrix for monkey F, step amplitude −10 deg. The gray level value in each pixel represents the correlation coefficient between the steering error at one moment in time, *θ(t)*, and the steering response at another moment in time, *r(t')*. The solid white line shows where *t* = *t'*.

As is apparent in Figure 8, the current steering response correlated with the steering error over time. While the exact structure of the steering correlation matrix changed with monkey and step amplitude, they all shared similar qualitative features: a negative region of correlation extended in time such that it runs parallel to (but above) the line of unity and a positive region of correlation extended in time such that it runs parallel to (but below) the line of unity. This structure provides some evidence as to the nature of the steering system. The region above the unity line corresponds to a negative correlation between the steering response and the steering error some time later. This is expected; if a monkey is turning toward the target faster than its mean turn velocity at a given time after a target step, than the steering error is going to be less than the average error for that target step at future time points. Conversely, if the monkey is turning slower than its mean turn velocity at a given time point after a target step, than the future steering error will likely be greater than the mean steering error after the given time. As time goes on the correlation weakens because the steering error has less dependence on the amplitude of the target step; instead, the trial by trial steering response integrated over time determines how much the steering error differs from the mean.

More interesting is the structure of the correlation below the line of unity in figure 8. This lobe of positive correlation corresponds to the situation when, at some time *t*, the steering error was larger than average, then some time later (at time *t'* = *t*+Δ*t*) the response tended to be more than average, indicating the system is compensating for the increased steering error Δ*t* seconds in the past. The positive region correlates steering responses to the steering error 250–350 ms before the response at a given time, giving us an approximate estimate of the lag Δ*t*. This positive region runs parallel to the unity line, indicating the steering error is sampled continuously at a constant lag during a steering response. This zone of maximum correlation is also similar, as one would expect, to the measured latency to step responses.

### Eye movements

When a primate fixates a target, information about the target location relative to the head can be recovered from the position of the eyes within their orbits [9], [10]. If steering behavior relies on eye position, we would expect the steering responses to follow the position of the eye from trial to trial. To test this hypothesis, we recorded the eye position of the monkeys as they performed our steering task. As one would expect, the eye movements following a target step start with a saccade to the new target location, followed by smooth pursuit of the target until the next time the target was stepped. However, when we compare the latency to the saccade to the latency to the initial steering response, we find the saccade latency fails to predict the response latency with any strength or significance (all p-values >0.05). This suggests that, at least in the earliest phases of the steering response, the position of the eye does not inform the steering response.

**Figure 9:**
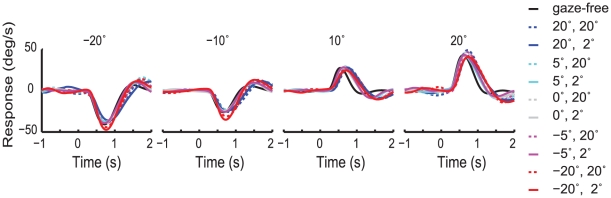
Mean step response by fixation condition and step size. Each trace shows the mean response to a given step size. Each fixation condition is represented by different traces.

Another test for the influence of eye movements is to force the monkey to fixate a target other than the steering target and ask if steering behavior is changed. We trained monkey F to perform the steering task while fixating an independent, stationary fixation point. As can be seen in Figure 9, the early shape of the steering response is generally unaffected by the inability to track the target with her eyes: the latency, slope and the peak of the mean responses are all extremely similar to the free viewing condition. As time passes, however, the response profile becomes both slower and larger in amplitude when compared to the gaze-free condition.

## Discussion

The main goal of this work was to create a laboratory setting where sensory cues were used to control a motor movement guiding a virtual trajectory. While the motor output was different from normal locomotor behavior, we believe this to be relevant to normal locomotion in many important ways. First, it is directly related to human steering behavior, which has been extensively studied and modeled. Deficiencies in human steering cause many unnecessary injuries and deaths each year, and understanding the mechanisms of steering at a physiological level would be of potentially great benefit. Secondly, while monkeys do not steer in their natural environment, they are clearly capable of learning the behavior rapidly and well (as are humans). So, we believe this indicates that essential sensory-motor control systems used in normal locomotion are in use in this task as well.

The visual cues we employed were a simplified version of the ones available in normal visually guided steering: target direction and optic flow. In our experiment, because of the trajectory-yoked view on the screen, the target direction was a necessary and prominent cue. Indeed, it would in principle be possible to perform the task without reference to the optic flow information at all. In related experiments, in which uncorrelated noise is added to both the flow and target position cues, we have been able to estimate the weights placed on each cue, and find that both cues are weighted approximately equally, in the case where equal amounts of noise are added to each (Egger and Britten, unpublished observations). These experiments are still in progress, and will be described in a forthcoming publication. However, these observations reassure us that the animals were likely to be using both cues in the version of the task used in the present work as well.

After training, each monkey was able to steer quickly and accurately toward a target. We found two principal results. First, responses to target steps were largely stereotyped, and their magnitudes scaled approximately linearly with increasing inputs. Secondly, during continuous epochs of steering, responses were continuously correlated with the magnitude of the preceding steering error, suggesting a sensory-motor feedback loop was engaged. Interestingly, the mean steering responses of the monkeys were very similar in appearance to human subjects in similar tasks [6].

While on average the monkeys performed excellently, there was some dispersion about the mean behavior on a trial by trial basis. We parameterized individual steering responses to determine if this dispersion was systematic and found that the steering behavior at various phases of the response was approximately Gaussian around each mean. This suggests that, instead of multiple steering strategies, a single steering strategy exists, but the system responsible for the behavior is subject to considerable noise. The exact source of this noise remains unclear, but it is likely the combination of noise in sensory estimates of the timing and size of the target steps and the transformation of that estimate into an appropriate motor response.

At least part of the noise at any time of the steering response is explained by the steering error 250–350ms before that time. Our experiments clearly show that variation in the steering response over time is correlated with the steering error in the immediate past. This suggests a system that continuously samples steering error, but there is some delay in the processing of sensory information and implementation of the motor response. Several researchers invoke a closed-loop model with a sensory-motor lag to explain similar biological movements [11], [12], [13], [14]. Combined with the observation that the time to the peak response depends on the relative gain of the system, a result predicted by closed loop models, we can be fairly confident the monkeys use a closed-loop strategy.

Our observations are in general agreement with previous research on steering behavior in humans. Several authors propose a model where the acceleration of a turn is a linear function of the instantaneous steering error plus a dampening term [2], [7]. However, our results suggest a lag not included in previous models. A likely source for this lag is the time required for the system to process visual information and to initiate a motor response. We cannot rule out other sources of delay, such as the time required to overcome the inertia of the hand and arm as they manipulate the joystick. In the future, we will develop a model of the steering system to help differentiate between these hypotheses.

One previous experiment [15] has reported data from monkeys trained on a steering task. The main focus in this paper was on the responses of neurons in the medial superior temporal area (MST), and not on the details of the behavior, as in the present work. While the traces shown do not make the time series evident, the authors state that the monkeys slowed as they approached the target, presumably to avoid overshooting. In our data, overshoots were conspicuous. The most likely explanation for this difference was in the much higher joystick gain we used (maximum deflection in their experiments produced a slew rate of 10 °/s, as opposed to 85–255 °/s in ours). Another possibility lies in differing reward strategies. Our monkeys were actively encouraged to steer rapidly by delivering their first reward immediately upon re-acquisition of the correct heading. In the Page and Duffy work, reward was delayed for one second. Lastly, it is possible that our inclusion of an explicit target position cue changed the behavioral dynamics. We believe this last possibility to be very unlikely, based on ongoing experiments in which we vary the relative reliability of the two cues; this has no apparent effect on the dynamics of steering (Egger and Britten, unpublished data).

The correlation between the current steering response and error ∼300ms before can only explain at most ∼60% of the variance in the response to a target step. The other sources of variation remain unknown. One possible source is the variation in the motor response. However, a major source is likely in the estimation of the steering error by the system. The Gaussian nature of the variation in the steering response suggests that several variables influence the steering response. Other studies have suggested that this is, in fact, the case [1], [2], [3], [5], [16]. In our paradigm, the target bearing, either relative to the observer or to room landmarks, as well as its position relative to the optic flow pattern, all provide cues for the monkey to use to estimate steering error. Our current set of experiments cannot address the relative weighting of these cues. However, one of the advantages of this paradigm is that many of these cues can be brought under experimental control so we can estimate their weighting. Future experiments will address the estimation of steering error and the relative strength of the many possible visual cues available for this task.

Gaze direction has assumed considerable prominence as a cue in steering, as well as in other visually guided behaviors (e.g., [17], [18]). However, it remains an open question as to whether gaze influences natural behavior through providing better scene information, or if gaze direction actually exerts direct, metric influence on subsequent movements. We tested the influence of eye position in two ways. First, we compared the timing of the saccades to the target to the latency of the response. We found no correlation, even though the saccades usually preceded the steering response. This indicates that, at least early in the steering response, the position of the eye was unimportant to the dynamics of the task. Second, we trained a monkey to perform the steering task while maintaining constant fixation on another visual target. Steering behavior was unaffected up to the time of the peak response. After the peak response, however, the steering behavior becomes slower and the error increases modestly. This result, which agrees with human data [17], is consistent with either interpretation, since the late stages of steering in our task involve smaller errors, and presumably require higher-resolution visual information. The absence of influence on the early stages of the task, however, strongly refutes the necessity of gaze changes for directing steering movements.

For many years, our lab and others have studied primate optic flow processing using purely perceptual tasks [19], [20], [21], [22], [23], [24], [25], [26], [27]. However, some have called into question the validity of these conclusions for active navigation [4], [28]. We are now in the unique position to determine if the results from single neuron studies generalize to a more active task. Further, since the similarities between human and monkey steering behaviors suggest both primates share a similar steering system, we can identify the neural systems necessary and sufficient for primate steering behavior, including that of humans.

## References

[1] Gibson JJ (1950). Perception of the visual world.

[2] Wilkie RM, Wann JP (2002). Driving as night falls: the contribution of retinal flow and visual direction to the control of steering.. Curr Biol.

[3] Wilkie RM, Wann JP (2005). The role of visual and nonvisual information in the control of locomotion.. J Exp Psychol Hum Percept Perform.

[4] Wilkie RM, Wann JP (2006). Judgments of path, not heading, guide locomotion.. J Exp Psychol Hum Percept Perform.

[5] Warren WH, Kay BA, Zosh WD, Duchon AP, Sahuc S (2001). Optic flow is used to control human walking.. Nat Neurosci.

[6] Hildreth EC, Beusmans JM, Boer ER, Royden CS (2000). From vision to action: experiments and models of steering control during driving.. J Exp Psychol Hum Percept Perform.

[7] Fajen BR, Warren WH (2003). Behavioral dynamics of steering, obstacle avoidance, and route selection.. J Exp Psychol Hum Percept Perform.

[8] Judge SJ, Richmond BJ, Chu FC (1980). Implantation of magnetic search coils for measurement of eye position: An improved method.. Vision Res.

[9] Hollands MA, Patla AE, Vickers JN (2002). “Look where you're going!”: gaze behaviour associated with maintaining and changing the direction of locomotion.. Exp Brain Res.

[10] Land MF, Tatler BW (2001). Steering with the head. the visual strategy of a racing driver.. Curr Biol.

[11] Sabes PN (2000). The planning and control of reaching movements.. Curr Opin Neurobiol.

[12] Churchland MM, Lisberger SG (2001). Experimental and computational analysis of monkey smooth pursuit eye movements.. J Neurophysiol.

[13] Reichardt W, Poggio T (1976). Visual control of orientation behaviour in the fly. Part I. A quantitative analysis.. Q Rev Biophys.

[14] Boeddeker N, Egelhaaf M (2005). A single control system for smooth and saccade-like pursuit in blowflies.. J Exp Biol.

[15] Page WK, Duffy CJ (2008). Cortical neuronal responses to optic flow are shaped by visual strategies for steering.. Cerebral Cortex.

[16] Wilkie R, Wann J (2003). Controlling steering and judging heading: retinal flow, visual direction, and extraretinal information.. J Exp Psychol Hum Percept Perform.

[17] Wilkie RM, Wann JP (2003). Eye-movements aid the control of locomotion.. J Vis.

[18] Mennie N, Hayhoe M, Sullivan B (2007). Look-ahead fixations: anticipatory eye movements in natural tasks.. Exp Brain Res.

[19] Britten KH, van Wezel RJA (1998). Electrical microstimulation of cortical area MST biases heading perception in monkeys.. Nature Neurosci.

[20] Royden CS, Banks MS, Crowell JA (1992). The perception of heading during eye movements.. Nature.

[21] Warren WH, Hannon DJ (1988). Direction of self-motion is perceived from optical flow.. Nature.

[22] Duffy CJ (1998). MST neurons respond to optic flow and translational movement.. J Neurophysiol.

[23] Paolini M, Distler C, Bremmer F, Lappe M, Hoffmann KP (2000). Responses to continuously changing optic flow in area MST.. J Neurophysiol.

[24] Stone LS, Perrone JA (1997). Human heading estimation during visually simulated curvilinear motion.. Vision Research.

[25] Heuer HW, Britten KH (2004). Optic flow signals in extrastriate area MST: comparison of perceptual and neuronal sensitivity.. J Neurophysiol.

[26] Zhang T, Heuer HW, Britten KH (2004). Parietal Area VIP Neuronal Responses to Heading Stimuli Are Encoded in Head-Centered Coordinates.. Neuron.

[27] Bradley DC, Maxwell M, Andersen RA, Banks MS, Shenoy KV (1996). Mechanisms of heading perception in primate visual cortex.. Science.

[28] Rushton SK, Harris JM, Lloyd MR, Wann JP (1998). Guidance of locomotion on foot uses perceived target location rather than optic flow.. Curr Biol.

